# Thermodynamic Driving Force of Hydrogen on Rumen Microbial Metabolism: A Theoretical Investigation

**DOI:** 10.1371/journal.pone.0161362

**Published:** 2016-10-26

**Authors:** Henk J. van Lingen, Caroline M. Plugge, James G. Fadel, Ermias Kebreab, André Bannink, Jan Dijkstra

**Affiliations:** 1 TI Food and Nutrition, Wageningen, The Netherlands; 2 Animal Nutrition Group, Wageningen University, Wageningen, The Netherlands; 3 Laboratory of Microbiology, Wageningen University, Wageningen, The Netherlands; 4 Department of Animal Sciences, University of California, Davis, Davis, California, United States of America; 5 Animal Nutrition, Wageningen UR Livestock Research, Wageningen, the Netherlands; The University of Akron, UNITED STATES

## Abstract

Hydrogen is a key product of rumen fermentation and has been suggested to thermodynamically control the production of the various volatile fatty acids (VFA). Previous studies, however, have not accounted for the fact that only thermodynamic near-equilibrium conditions control the magnitude of reaction rate. Furthermore, the role of NAD, which is affected by hydrogen partial pressure (*P*_H_2__), has often not been considered. The aim of this study was to quantify the control of *P*_H_2__ on reaction rates of specific fermentation pathways, methanogenesis and NADH oxidation in rumen microbes. The control of *P*_H_2__ was quantified using the thermodynamic potential factor (*F*_T_), which is a dimensionless factor that corrects a predicted kinetic reaction rate for the thermodynamic control exerted. Unity *F*_T_ was calculated for all glucose fermentation pathways considered, indicating no inhibition of *P*_H_2__ on the production of a specific type of VFA (e.g., acetate, propionate and butyrate) in the rumen. For NADH oxidation without ferredoxin oxidation, increasing *P*_H_2__ within the rumen physiological range decreased *F*_T_ from unity to zero for different NAD^+^ to NADH ratios and pH of 6.2 and 7.0, which indicates thermodynamic control of *P*_H_2__. For NADH oxidation with ferredoxin oxidation, increasing *P*_H_2__ within the rumen physiological range decreased *F*_T_ from unity at pH of 7.0 only. For the acetate to propionate conversion, *F*_T_ increased from 0.65 to unity with increasing *P*_H_2__, which indicates thermodynamic control. For propionate to acetate and butyrate to acetate conversions, *F*_T_ decreased to zero below the rumen range of *P*_H_2__, indicating full thermodynamic suppression. For methanogenesis by archaea without cytochromes, *F*_T_ differed from unity only below the rumen range of *P*_H_2__, indicating no thermodynamic control. This theoretical investigation shows that thermodynamic control of *P*_H_2__ on individual VFA produced and associated yield of hydrogen and methane cannot be explained without considering NADH oxidation.

## Introduction

Carbohydrates ingested by ruminants are degraded into monomers by action of rumen microbial enzymes and subsequently fermented to products such as volatile fatty acids (VFA) and alcohols. The most common pathway of hexose metabolism in rumen microbes is glycolysis, which yields two equivalents of pyruvate, ATP and NADH. The NADH, a cofactor carrying electrons, needs to be oxidized back to NAD^+^ to keep the glycolysis possible and to maintain further metabolic steps of the overall microbial metabolism that depend on pyruvate [1, 2]. The oxidation of NADH to NAD^+^ may be directly coupled to the product formation from pyruvate that follows glycolysis. Production of butyrate couples the oxidation of NADH to the reduction of acetoacetyl-CoA as well as crotonyl-CoA [3]. Various fermentative micro-organisms are also able to convert pyruvate into ethanol, lactate or succinate [4], which results in direct oxidation of NADH. Acetate is quantitatively the main VFA in the rumen, but its production from pyruvate is not directly coupled to the oxidation of NADH. In this case, NADH is oxidized via H_2_ production, which is thermodynamically inhibited at elevated hydrogen partial pressure (*P*_H_2__). Oxidation of NADH may be thermodynamically feasible by coupling it to the oxidation of reduced ferredoxin [5]. Many methanogenic archaea utilize H_2_ to reduce CO_2_ to CH_4_. This keeps *P*_H_2__ at a low level, which enables NADH oxidation in bacteria that are not able to directly couple NADH oxidation to reduction of metabolites [4].

Multiple estimates of rumen VFA (e.g., acetate, propionate, butyrate and other) production from feed substrate have been reported in literature based on factors including type of organic matter fermented and type of diet [6]. Such estimates are required in rumen models to predict the amount and type of VFA entering the intermediary metabolism of ruminants. Another application of these estimates is the prediction of enteric CH_4_ production, which is of interest in terms of the environment. Accuracy of predicted CH_4_ emission by the model used by Bannink et al. [7] appeared to be mostly affected by the error in the representation of the molar proportion at which individual VFA are produced. Reducing this error contributes to more adequate prediction of enteric CH_4_ emission [8]. A recent metabolic model of mixed culture fermentation [9] represents how incorporation of thermodynamically controlled cofactor dynamics may improve the prediction of end products such as VFA from glucose fermentation.

Thermodynamic control of rumen fermentation pathways by *P*_H_2__ has been investigated to explain variation in observed VFA concentrations [10]. Thermodynamic control is often evaluated by Gibbs energy change (Δ*G*). Negative values of Δ*G* indicate a reaction to proceed in the forward direction, positive values in the reverse direction, and Δ*G* = 0 indicates equilibrium. Using Δ*G*, it has been explained that increased concentrations of H_2_ result in a shift to pathways forming propionate at the expense of acetate as an alternative way of accepting electrons to H_2_-forming pathways because the latter become thermodynamically less favorable [11]. Reaction rates of fermentation pathways have been prescribed by setting the quotient of kinetic rate constants for the forward and reverse reaction equal to the thermodynamic equilibrium constant [10, 12]. However, the quotient of the rate laws for reverse and forward reaction does not necessarily reflect the stoichiometry of a reaction and is not in general similar to the thermodynamic equilibrium constant. Besides, classical thermodynamic functions such as Δ*G* have no implications for the magnitude of reaction rate, except for near-equilibrium situations [13], and may not rigorously account for the thermodynamic driving force on reaction [14]. Furthermore, various investigations on the control of *P*_H_2__ on rumen fermentation have ignored the role of NAD, or have mentioned it without quantifying the redox state as affected by varying *P*_H_2__ (e.g., [1, 2, 11, 12]). The aim of the present study is to quantify the thermodynamic effect of *P*_H_2__ on the reaction rate of specific fermentation pathways, NADH oxidation and methanogenesis in the rumen.

## Methods

### Metabolic pathways

Glucose can be fermented via various pathways depending on the microbial diversity and the conditions in the rumen environment. To quantify the effect of *P*_H_2__ on reaction rates, five rumen glucose fermentation pathways each yielding different VFA, three H_2_-dependent interconversions of VFA (viz. acetate to propionate, propionate to acetate and butyrate to acetate), oxidation of NADH with and without reduced ferredoxin oxidation, and methanogenesis were considered (Table 1). Selected reactions focus on formation of VFA and have been taken from ref. [3] for reactions b, j and k; ref. [4] for reactions a, g, h and i; ref. [15] for reaction f; ref. [16] for reaction c when butyrate is produced via the kinase route; and ref. [17] for reactions c when butyrate is produced via the CoA-transferase route, and reactions d and e. Conversions of acetate to butyrate, butyrate to propionate and propionate to butyrate are discussed, but the effect of *P*_H_2__ on reaction rate is not shown because these conversions do not yield any H_2_ or have limited physiological significance. Glucose fermentation reactions in Table 1 are ordered following the stoichiometry of H_2_ formation. The number of NADH oxidized with H_2_ formation for the interconversion reactions were obtained considering reactions f and g as linear combinations of reactions a and d, and reaction h as a linear combination of reactions a and c. Various other cofactors are involved in the microbial degradation of glucose as well, but only NAD is involved in both the glycolysis and in further metabolic pathways of pyruvate to VFA or other fermentation products. The redox state of this cofactor explains the shift in pathways of glucose fermentation and therefore the focus is on oxidation of NADH. Besides being involved in NADH oxidation via confurcation, ferredoxin is involved in the production of acetate and butyrate, which explains why the H_2_ yield reported for metabolic pathways in Table 1 may not be equal to the number of NADH oxidized with H_2_ formation.

**Table 1:** Possible glucose fermentation pathways to VFA (Ac^-^, Pr^-^ and Bu^-^ for acetate, propionate and butyrate, respectively), volatile fatty acid (VFA) interconversions, hydrogenase-catalyzed NADH oxidation and methanogenesis in the rumen and their yield of ATP (Y_ATP_), number of NADH to be oxidized with H_2_ formation (Y_NADH_, mol per mol of glucose), the standard reaction Gibbs energy (Δ*G*^*o*^ in kJ⋅mol^−1^, standardized to concentrations of 1 M, pH of 0, gas pressure of 1 bar) adjusted to 312K, and the average stoichiometric number *χ*.

Microbial conversion				Y_ATP_	Y_NADH_	Δ*G*^*o*^	*χ*
**Glucose fermentation**							
a)	C_6_H_12_O_6_ + 4H_2_O	→	2 Ac^−^ + ${\text{2HCO}}_{3}^{-}$ + 4H_2_ + 4H^+^	4	2	-52	4
b)	C_6_H_12_O_6_ + 2.67 H_2_O^a^	→	0.67 Ac^−^ + 0.67 Bu^−^ + ${\text{2HCO}}_{3}^{-}$ + 3.33H^+^ + 2.67H_2_	3.33	0.67	-111	3.33
c)	C_6_H_12_O_6_ + 2H_2_O^b^	→	Bu^−^ + ${\text{2HCO}}_{3}^{-}$ + 2H_2_ + 3H^+^	3	0	-138	3
d)	C_6_H_12_O_6_ + H_2_O^c^	→	Ac^−^ + Pr^−^ + ${\text{HCO}}_{3}^{-}$ + H_2_ + 3H^+^	3.67	0	-159	3
e)	C_6_H_12_O_6_ ^d^	→	0.67 Ac^−^ + 1.33 Pr^−^ + $\text{0}{\text{.67HCO}}_{3}^{-}$ + 2.67H^+^	2.67	-0.67	-196	2.67
**VFA interconversion**							
f)	Ac^−^ + ${\text{HCO}}_{3}^{-}$ + H^+^ + 3H_2_	→	Pr^−^ + 3H_2_O	0	-2	-113	1
g)	Pr^−^ + 3H_2_O	→	Ac^−^ + ${\text{HCO}}_{3}^{-}$ + H^+^ + 3H_2_	0.33	2	113	2
h)	Bu^−^ + 2H_2_O	→	2 Ac^−^ + H^+^ + 2H_2_	0.33	2	86	2
**Cofactor oxidation**							
i)	NADH + H^+^	→	NAD^+^ + H_2_	0	NA	-25	1
j)	NADH + ${\text{Fd}}_{\text{RED}}^{2-}$ + 3H^+^	→	NAD^+^ + Fd_OX_ + 2H_2_	0	NA	-102	2
**Methanogenesis**							
k)	${\text{HCO}}_{3}^{-}$ + H^+^ + 4H_2_	→	CH_4_ + 3H_2_O	1.5 or 0.5^e^	0	-172	2

*^a^*Butyrate production via the kinase route

*^b^*Either for butyrate production via the kinase route or a linear combination of reaction a) and 2Ac^−^ + 2C_6_H_12_O_6_ + 2H_2_O → 3Bu^−^ + ${\text{4HCO}}_{3}^{-}$ + 2H_2_ + 5H^+^ for butyrate production via the CoA-transferase route

*^c^*Propionate production via succinate

*^d^*Propionate production via lactate

*^e^*for archaeal species with and without cytochromes

Moreover, as has been compared to the formation of propionate at the expense of acetate, reductive acetogenesis may be a potential alternative H_2_ sink to methanogenesis in the rumen [18], but will not be considered in the present investigation. Although this conversion is associated with carbon turnover and is common in environments such as the human colon [17] and foregut of kangaroos and wallabies [19], acetogenic bacteria in the rumen have been hypothesized to be unable to compete for H_2_ with the methanogens (e.g., [20]). Unless mentioned otherwise, respiration was assumed not to be occurring within the rumen microbiome.

### Thermodynamic potential factor

The thermodynamic control on rates of rumen fermentation pathways was quantified using the thermodynamic potential factor (*F*_T_) as derived by Jin and Bethke [14]. This factor modifies commonly used rate laws and makes them thermodynamically consistent by accounting for the difference between the energy available through fermentation and the energy conserved. The energy available through fermentation is calculated from the ratio of reactants and products, which is associated with the progress of the forward and reverse direction of a reaction. A rate law that accounts for the forward as well as the reverse direction of a reaction is thermodynamically consistent and may be represented as:

$$\begin{array}{c} r=k\left[X\right]\frac{\left[S\right]}{\left[S\right]+K_{S}}F_{T}, \end{array}$$ (1)

with the kinetic rate constant *k*, the microbial biomass concentration [X], the substrate concentration [S] and the half-saturation constant *K*_*S*_. Kinetic rate laws, however, are often developed assuming that a large thermodynamic force drives a metabolic reaction forward. Under this condition, kinetic rate laws do not need to be corrected with any factor like *F*_T_. This assumption is reasonable when the environment is rich in chemical energy, that is where the metabolic reaction is far from equilibrium. The *F*_T_ is mathematically represented as:

$$\begin{array}{c} F_{T}=1-\text{exp}\left(-\frac{\Delta G_{A}-\Delta G_{C}}{\chi RT}\right), \end{array}$$ (2)

where Δ*G*_*C*_ is the energy conserved (J⋅mol^-1^), which is commonly determined from the number of ATP produced times the Gibbs energy of phosphorylation (Y_ATP_⋅Δ*G*_*P*_; Δ*G*_*P*_ is approximated by 44 kJ⋅(mol ATP)^-1^ for rumen microbes in the present study); Δ*G*_*A*_ is the energy available through fermentation (J⋅mol^-1^); *χ* is the average stoichiometric number representing the number of times elementary steps of product formation occurs relative to the main reactant; *R* the gas constant (8.31 J⋅mol^-1^⋅K^-1^); *T* the temperature (312K in the rumen); this makes *F*_T_ dimensionless by definition. For Δ*G*_*A*_ ≫ Δ*G*_*C*_ and common values of *T* and *χ*, *F*_T_ approaches 1 (also designated as unity), and the net reaction rate is 100% of the forward rate, and *F*_T_ can be neglected in determining rates of reaction in microbial metabolism. When Δ*G*_*A*_ approaches Δ*G*_*C*_, the forward and reverse reaction approach equilibrium, which is reflected in *F*_T_ approaching zero. For Δ*G*_*A*_ < Δ*G*_*C*_, *F*_T_ becomes negative, suggesting that a reaction net proceeds in the reverse direction; for Δ*G*_*A*_ ≪ Δ*G*_*C*_, and common values of *T* and *χ*, *F*_T_ approaches –∞ suggesting that the forward reaction is even negligibly small compared to the reverse reaction. Negative *F*_T_ may not be useful for prediction of reaction rate since common rate laws of such as the Monod equation are not used for reactions that overall proceed in the reverse direction. At a microbial level, a reverse reaction would consume energy rather than contribute to a cell’s energy budget, which is not enzymatically supported and the metabolism may stop.

The Δ*G*_*A*_ is further specified as:

$$\begin{array}{c} \Delta G_{A}=-\Delta G^{o}-RT\;\text{ln}\;Q, \end{array}$$ (3)

with Δ*G*^*o*^ the standard reaction Gibbs energy, and *Q* the reaction quotient, which is:

$$\begin{array}{c} Q=\prod_{J}a_{J}^{\nu_{J}}, \end{array}$$ (4)

where *a*_J_ denotes the concentration of substance J, and *ν*_J_ its corresponding stoichiometric number in the chemical equation, which is positive for products and negative for reactants. Substituting Eqs 3 and 4 into Eq 2 yields:

$$\begin{array}{c} F_{T}=1-Q^{\chi^{-1}}\;\text{exp}\left(\frac{\Delta G^{o}+\Delta G_{C}}{\chi RT}\right). \end{array}$$ (5)

Substances that are in the gaseous state under rumen conditions are represented in partial pressure instead of aqueous concentrations; water activity is assumed to be 1 and omitted from the reaction quotient in any case. To illustrate, *F*_T_ for the glucose to acetate conversion (reaction a, Table 1) by substituting into Eq 5 gives:

$$\begin{array}{c} F_{T}=1-{\left[{\text{Ac}}^{-}\right]}^{0.5}{\left[{\text{HCO}}_{3}^{-}\right]}^{0.5}P_{H_{2}}\left[H^{+}\right]{\left[C_{6}H_{12}O_{6}\right]}^{-0.25}\;\text{exp}\left(\frac{-52\cdot 10^{3}+4\cdot 44\cdot 10^{3}}{4\cdot 8.31\cdot 312}\right). \end{array}$$ (6)

### Reaction specific energy conservation and elementary reaction steps

In anaerobic fermentation, ATP is mostly produced by substrate level phosphorylation, but some electron transport phosphorylation may take place during fermentations [16, 21]. Reaction steps associated with electron transport phosphorylation include fumarate reduction in the pathways of pyruvate to propionate, crotonyl-CoA reduction in the pathway of acetyl-CoA to butyrate, and the oxidations of succinate and butyryl-CoA in the syntrophic conversions of propionate to acetate and butyrate to acetate. Yield of ATP (shown in Table 1 for every reaction considered) was assumed to be 2 for the common pathway of glucose to 2 pyruvate, and 2, 1.33 and 1 for the conversion of 2 pyruvate into 2 acetate, 2 propionate and 1 butyrate, respectively [4, 9]; 0.33 for the oxidations of propionate and butyrate to acetate [4]; 0 for the reduction of acetate and ${\text{HCO}}_{3}^{-}$ to propionate [22]; and 1.5 or 0.5 per equivalent of CH_4_ produced by archaeal species with and without cytochromes [3, 23]. Since ATP was described to be generated by substrate level phosphorylation only for *Clostridium pasteurianum* [3], and uncertain ATP yield from electron transport phosphorylation was predicted for rumen *Butyrivibrios* [16], reference values of ATP yield used in the present study may be subject to revision.

For microbial catabolism, likely rate-determining steps may be substrate level phosphorylation during fermentation, proton translocation, substrate activation or electron transfer to extracellular electron acceptors [14]. For rumen glucose fermentation, the rate-determining step was chosen to be equal to the ATP yield from substrate level phosphorylation, which results in *χ* equal to 4, 3.33, 3, 3 and 2.67 for reactions a to e (Table 1). When assuming reactivity of NADH oxidation to be dominated by hydride transfer [24], the rate-determining step occurs only once per equivalent of NADH oxidized, indicating *χ* = 1 for NADH oxidation without ferredoxin oxidation. Although various aspects of hydrogenase-catalyzed cofactor oxdation require further clarification, a hydride intermediate may also be formed in the oxidation of reduced ferredoxin [25]). NADH oxidation via electron confurcation (i.e., combining electrons from two dissimilar donors to generate a single product such as H_2_) would then be associated with two hydrides intermediates, indicating *χ* = 2 for NADH oxidation with ferredoxin oxidation. The rate-determining step for the reduction of acetate and ${\text{HCO}}_{3}^{-}$ (reaction f, Table 1) was assumed to be the activation of acetate to acetyl phosphate. This activation occurs once per equivalent of acetate, which makes *χ* = 1. The butyryl-CoA and succinate oxidations are the energetically most demanding steps in the overall pathways of butyrate and propionate fermentation [4], where electron transfer was taken as the rate-determining step. Two electrons are transferred for both the oxidation of butyryl-CoA and succinate, which indicates *χ* = 2 for both conversions. The rate-determining steps in methanogenesis, with and without the involvement of cytochromes, were assumed to be the methyltetrahydromethanopterin-coenzyme M methyltransferase and the reduction of the disulfide of coenzymes B and M, respectively. Both steps are coupled to the translocation of two sodium ions [23], which occurs once per equivalent of CH_4_ produced, indicating *χ* = 2.

### Continuous input variables and uncertainty of *F*_T_

For reactions a to h and k, concentrations were 1 mM hexose, 60 mM acetate, 20 mM propionate, 12.5 mM butyrate and 40 mM bicarbonate, 0.25 bar partial pressure of CH_4_ and pH was equal to 6.45; for reaction j, Fd_RED_^2-^/Fd_OX_ was equal to 9. Values for Δ*G*^*o*^ of fermentation pathways and standard redox potentials of cofactors were taken from refs. [3, 10]. Values of Δ*G*^*o*^ of metabolite formation were adjusted to rumen temperature using the Van’t Hoff equation (e.g., [26]).

The uncertainty of *F*_T_ to variation in inputs other than *P*_H_2__ was assessed for the five glucose fermentation pathways, the three VFA interconversions and methanogenesis (Table 1). Ten thousand different samples were drawn randomly from uniform distributions for glucose, acetate, propionate, butyrate concentrations, pH, *P*_CO_2__, *P*_CH_4__ and Δ*G*_*P*_ ranging from 0.1 to 2.0 mM, 35 to 90 mM, 7 to 30 mM, 5 to 21 mM, 5.7 to 7.2, 0.35 to 0.80 bar, 0.15 to 0.35 bar and 35 to 50 kJ⋅mol^-1^, respectively. For completeness, proton concentrations were calculated from pH and ${\text{HCO}}_{3}^{-}$ concentrations were calculated using the Henderson-Hasselbalch equation (e.g., [27]).

Uncertainty of *F*_T_ approaches zero when *F*_T_ approaches unity. If *F*_T_ of a specific reaction deviated from unity for the range of *P*_H_2__ considered, a 95% confidence interval of *F*_T_ was calculated for 10 values of *P*_H_2__ for which *F*_T_ was close to zero at the previously mentioned fixed concentrations. Values of *P*_H_2__ increased exponentially in steps according to *P*_*H*_2_, *n*_ = *a*⋅*b*^*n*−1^, where *a* is the start value, *b* is the factor by which *P*_*H*_2_, *n*_ increases per step, and *n* runs from 1 to 10 for the number of steps. The exact values of *a* and *b* were chosen based on the visual representation of the uncertainty by the error bar. Applying this, the uncertainty of *F*_T_ was assessed for *P*_H_2__ at {2.00⋅10^−5^, 2.60⋅10^−5^, …, 2.12⋅10^−4^} bar for methanogenesis yielding 0.5 ATP, {6.00⋅10^−4^, 8.10⋅10^−4^, …, 8.94⋅10^−3^} bar for methanogenesis yielding 1.5 ATP, {7.70⋅10^−5^, 9.63⋅10^−5^, …, 5.74⋅10^−4^} bar for acetate to propionate conversion, {5.00⋅10^−6^, 6.50⋅10^−6^, …, 5.30⋅10^−5^} bar for propionate to acetate conversion, and {1.95⋅10^−6^, 2.93⋅10^−6^, …, 7.50⋅10^−5^} bar for butyrate to acetate conversion. The actual ranges of the 95% confidence intervals of *F*_T_ depends on metabolite concentrations and values of Δ*G*^*o*^, Δ*G*_*C*_ and *χ*, explicitly shown for the particular conversion of glucose into two equivalents of acetate (Eq 6). Eq 6 also shows the nonlinearity of *F*_T_ to its input, which makes the 95% confidence intervals asymmetric.

Calculation of the 95% confidence intervals of *F*_T_ at discrete values of *P*_H_2__ and plotting of *F*_T_ as a function of *P*_H_2__ was performed in R statistical software. Code is provided as supporting information (S1–S3 Files).

## Results and Discussion

### Glucose fermentation and NADH oxidation

**Fig 1:**
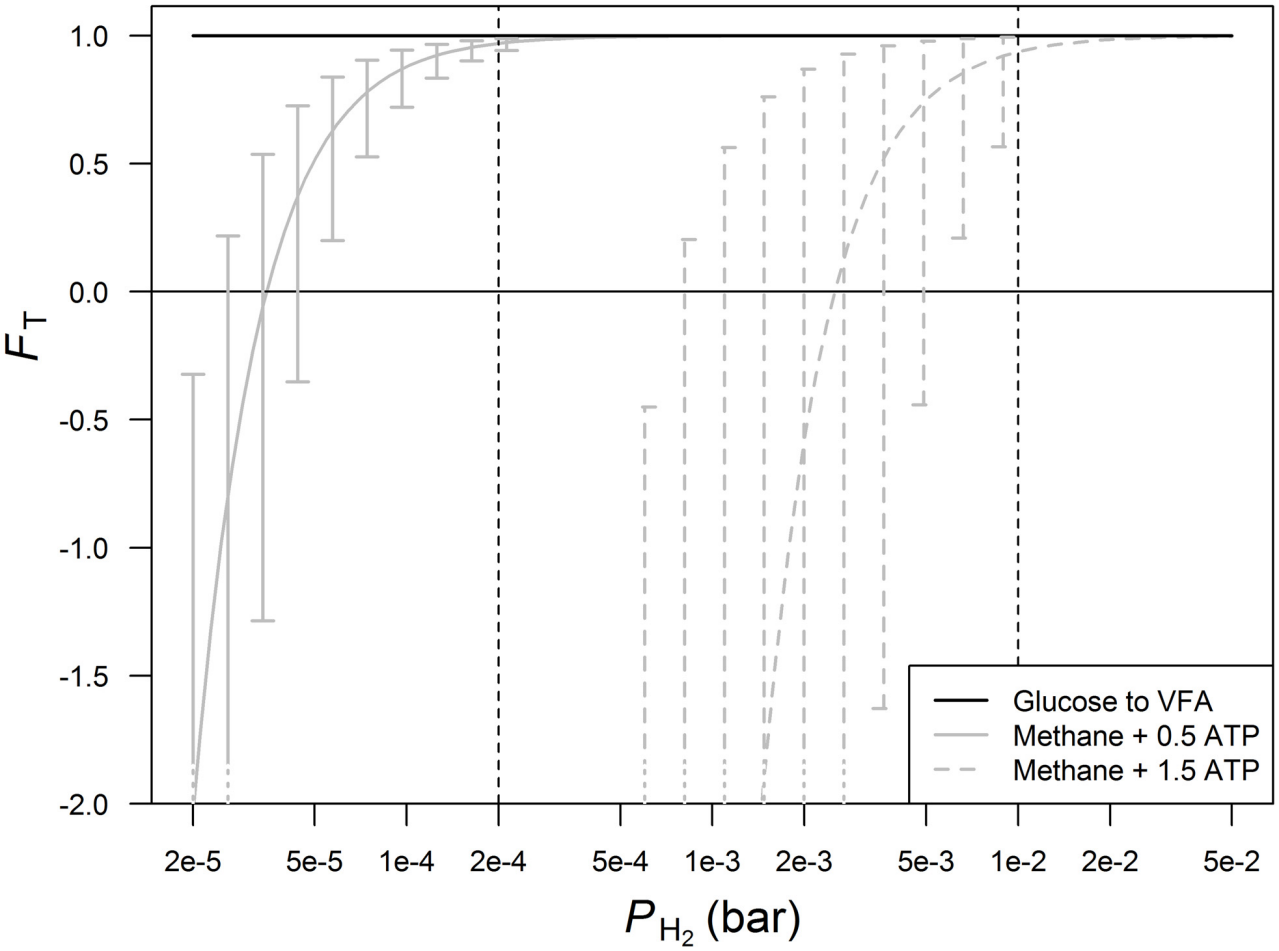
Thermodynamic potential factor (*F*_T_) as a function of *P*_H_2__ for glucose fermentation pathways and methanogenesis. The black line for glucose to VFA is valid for the reactions a to d (yielding acetate, propionate or butyrate), the solid and dotted gray lines represent methanogenesis with 0.5 and 1.5 mol of ATP per mol of CH_4_, respectively; a more detailed description of the glucose fermentation pathways to VFA and methanogenesis is given in Table 1. Confidence intervals represent uncertainty of *F*_T_ to variation in inputs other than *P*_H_2__. Vertical lines demarcate the rumen physiological range of *P*_H_2__. A log scale is used to plot the x-axis.

The *F*_T_ for the fermentation pathways a to d (Table 1) did not deviate from unity for *P*_H_2__ between 2⋅10^−5^ and 5⋅10^−2^ bar and had zero uncertainty (Fig 1), which is inherent to *F*_T_ approaching unity. This indicates these fermentation reactions proceed far from thermodynamic equilibrium and implies no inhibition on reaction rates since *P*_H_2__ in the rumen varies between 2⋅10^−4^ and 1⋅10^−2^ bar [2]. No *F*_T_ curve is shown for the conversion of glucose into 0.67 equivalents of acetate and 1.33 equivalents of propionate because it does not involve H_2_. The actual value of *F*_T_ for this conversion also yielded unity (result not shown) and indicates no thermodynamic inhibition of this fermentation pathway under the conditions assumed and range of *P*_H_2__ considered. In this investigation, we assumed an ATP yield of 3 per equivalent of butyrate if only substrate level phosphorylation takes place. Accounting for electron transport phosphorylation as well would predict an ATP yield of ∼ 4.5 per equivalent of glucose [16]. Production of propionate via succinate has also been mentioned to yield 4 ATP per equivalent of glucose [28]. Adjusting Δ*G*_*C*_ of reactions associated with propionate and butyrate to these higher yields of ATP still did not make *F*_T_ deviate from unity for the considered range of *P*_H_2__ (result not shown).

Absence of thermodynamic inhibition for any of the glucose fermentation pathways is not in line with conclusions drawn previously [11], where the conversion of glucose into VFA was considered to be directly affected by the level of H_2_. For common values of *χ* and *T*, *F*_T_ approaches unity when Δ*G*_*A*_ ≫ Δ*G*_*C*_, representing the far-from-equilibrium situation. This applies to the glucose fermentation pathways considered indicating that Δ*G* cannot be used as a measure of reaction rate for these reactions. This is in accordance with the fact that classical thermodynamic functions such as Δ*G* have no implications for magnitude of reaction rate, except for near-equilibrium situations [13]. Only a difference between Δ*G*_*A*_ and Δ*G*_*C*_ closer to zero than approximately –20 kJ⋅mol^-1^, which may be the cutoff for near-equilibrium, makes *F*_T_ deviate from unity. Additional evidence for Δ*G* ≈ −20*kJ*⋅mol^-1^ as a cutoff value for inhibited progress of microbial metabolism is given by Schink [29] who assumed a heat loss of about 20 kJ⋅mol^-1^ for irreversible metabolic processes that generate ATP. However, it was experimentally shown that syntrophic bacteria metabolize up to a zero difference between Δ*G*_*A*_ and Δ*G*_*C*_ [30], which corresponds to *F*_T_ = 0.

**Fig 2:**
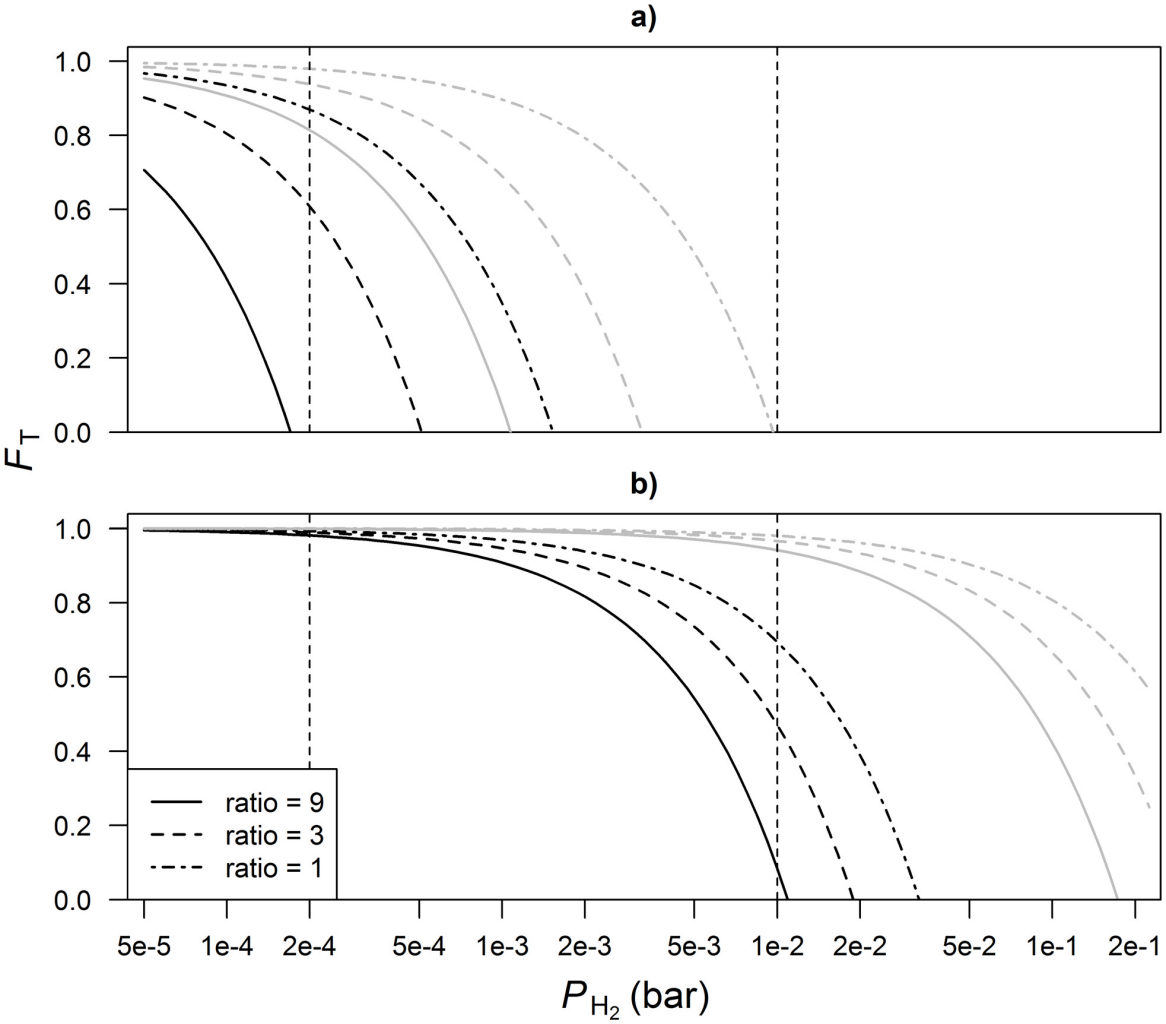
Thermodynamic potential factor (*F*_T_) as a function of *P*_H_2__ for a) NADH oxidation without ferredoxin oxidation and b) NADH oxidation with ferredoxin oxidation and the Fd_RED_^2-^ to Fd_OX_ ratio constant at 9. Line type represents NAD^+^ to NADH ratio and line color represents intracellular pH equal to 6.2 (gray) and 7.0 (black). Vertical lines demarcate the rumen physiological range of *P*_H_2__. A log scale is used to plot the x-axis.

The *F*_T_ for NADH oxidation without reduced ferredoxin oxidation decreased to zero upon an increase of *P*_H_2__ from 2⋅10^−4^ to 1⋅10^−2^ bar, whereas *F*_*T*_ < 1 may already be obtained at *P*_*H*_2__ < 5⋅10^−5^ bar for a high NAD^+^ to NADH ratio and pH = 7.0 (Fig 2a). The *F*_T_ for NADH oxidation with reduced ferredoxin oxidation decreased to zero at *P*_*H*_2__ > 1⋅10^−2^ bar, whereas *F*_*T*_ < 1 may already be obtained at *P*_*H*_2__ > 2⋅10^−4^ bar when pH = 7.0 (Fig 2b). The actual value of *F*_T_ depends on pH and NAD^+^ to NADH ratio. Partial pressure of H_2_ and intracellular pH of microbes in the rumen are assumed to vary between 2⋅10^−4^ and 1⋅10^−2^ bar [2], and 6.2 and 7.0 [31], respectively. Shortly after new feed enters the rumen, the rate of fermentation will increase, which results in a high *P*_H_2__ [32] and a low pH; whereas during fasting, *P*_H_2__ will be low and pH high. In an experimental study in which the effects of starch type and level on rumen fermentation were evaluated [33], the lowest acetate to propionate ratio was observed at 2 h after feeding, whereas the lowest pH was observed at 4 h after feeding. Achieving the lowest acetate to propionate ratio before the lowest pH may suggest that after feed consumption the increase in *P*_H_2__ occurs faster than the decrease in pH. This indicates that elevated *P*_H_2__ thermodynamically inhibits NADH oxidation shortly after feeding, but this is compensated by decreased pH later.

Although effects of the redox state of ferredoxin on the thermodynamic inhibition of NADH oxidation are not explicitly shown, ferredoxin is reduced during fermentation and the Fd_RED_^2-^ to Fd_OX_ ratio, which was assumed to be 9, may increase in response to increased metabolism shortly after ingestion of feed. If an increased Fd_RED_^2-^ to Fd_OX_ ratio applies to rumen bacteria, the inhibition of NADH oxidation is potentially alleviated. To evaluate this alleviation, the solid, dashed and dot-dashed lines in Fig 2b may, alternative to keeping the Fd_RED_^2-^ to Fd_OX_ constant at 9 and NAD^+^ to NADH ratios of 9, 3 and 1, correspond to keeping the NAD^+^ to NADH ratio constant at 9 and Fd_RED_^2-^ to Fd_OX_ ratios of 9, 27 and 81, respectively. This implies that the value of *F*_T_ is closer to 1 for more reduced ferredoxin, which weakens the thermodynamic force that inhibits NADH oxidation. Since ferredoxin is involved in the pathway from pyruvate to acetate and butyrate only and not in the glycolysis, whereas NAD may be involved in both pathways, the NAD^+^ to NADH ratio may change more rapidly after feeding than the Fd_RED_^2-^ to Fd_OX_ ratio. Inhibition of NADH oxidation may therefore occur shortly after feeding, but may be compensated later. Nonetheless, the present study demonstrates that the mechanism of NADH oxidation is critical for the magnitude of its inhibition; the inhibition of NADH oxidation is also determined by the thermodynamic state of the rumen with *P*_H_2__ and intracellular pH both being important determinants.

The NAD^+^ to NADH ratio is sometimes assumed to be in thermodynamic equilibrium with *P*_H_2__ [34], or in other words, *F*_T_ is assumed zero for any value of *P*_H_2__. For rumen bacteria incapable of confurcation this implies the NAD^+^ to NADH ratio is ≥ 9 at *P*_H_2__ = 2⋅10^−4^ and ≤ 1 at *P*_H_2__ = 1⋅10^−2^ bar (Fig 2a); for rumen bacteria in which confurcation does take place this implies the NAD^+^ to NADH ratio is ≥ 9 for *P*_*H*_2__ ≤ 1⋅10^−2^ bar (Fig 2b). The NAD^+^ to NADH ratio was reported to be 1.4 to 2.6 in rumen microbes [35], 1.1 to 2.7 for *Escherichia coli* [36], and was reported to be < 9 in living cells [3]. These ratios largely fall within the range of our prediction but tend to be at the edge of physiological feasibility and the NAD^+^ to NADH ratio in bacteria incapable of confurcation may underestimated at elevated *P*_H_2__. Although many anaerobic and syntrophic bacteria contain enzymes that catalyze electron confurcation, it is not evident whether many of the bacteria belonging to the core community in the rumen (e.g., *Prevotella*, *Fibrobacter*, *Ruminococcaceae*, *Bacteroidales*; [37]) employ this mechanism. *Ruminococcus albus* 7 that is part of the rumen core community employs this mechanism [38]. In this strain, genes encoding for the hydrogenase enzyme involved in electron confurcation had a similar transcript abundance in mono- and biculture. In contrast, genes encoding for a different hydrogenase that reduces protons to molecular hydrogen using reduced ferredoxin only was 90-fold upregulated in mono- compared to biculture [39]. This suggests that the confurcating hydrogenase functions in central metabolism regardless of external *P*_H_2__. Nonetheless, increased propionate to acetate ratios [33] and production of lactate being reported in response to feeding [40] may indicate these latter two ways of NADH oxidation are important alternatives for ferredoxin dependent oxidation of NADH. Direct evidence of how these mechanisms are applied by rumen bacteria is lacking however.

Given that the NAD^+^ to NADH ratio becomes less than or equal to 1 (Fig 2a), glycolytic reactions may be downregulated. Glycolytic activity of *Caldicellulosiruptor saccharolyticus* was found not to be completely inhibited at a NAD^+^ to NADH ratio equal to 1 [41], which may allow metabolic activity at ratios < 1. Nonetheless, highly reduced NAD is reconditioned to more oxidized NAD by the upregulation of the production of metabolites such as lactate and ethanol, as explained for gut microbiota [42]. This upregulation may take place in addition to increased proportions of propionate production. However, the production of lactate and ethanol is less favorable for microbial growth because conversion of pyruvate to either lactate or ethanol does not yield any ATP, unlike the conversion of pyruvate to acetate or butyrate, and to propionate via succinate. Another way in which bacteria may control *P*_H_2__ and the redox state of NAD in the rumen environment is the production of formate. Formate may be produced when pyruvate is converted to acetyl-CoA as an alternative for the oxidation of reduced ferredoxin [4]. Formate can be converted to H_2_ and CO_2_, but may also be directly used for CH_4_-production by methanogens [43]. In the latter case, no H_2_ is produced and the synthesis of formate serves as a potential mechanism to maintain low *P*_H_2__ [44].

The present theoretical exercise indicates that, in the rumen, *P*_H_2__ does not directly control the glucose fermentation pathways. However, depending on mechanism and pH, *P*_H_2__ does thermodynamically control NADH oxidation, which influences VFA production. NAD^+^ to NADH ratio as a key controller of fermentation end product formation is widely recognized in literature (e.g., [45, 46]). When the NAD^+^ to NADH ratio is low, the metabolism needs to yield more reduced products to oxidize NADH [41, 42]. Production of butyrate and propionate from reactions c and d both oxidize all NADH obtained from glycolysis back to NAD^+^ (Table 1) but does not explain why elevated propionate but no elevated butyrate is found at increased *P*_H_2__. A difference between these pathways is the H_2_ yield of 2 and 1 equivalents per equivalent of glucose from reaction c and d, respectively. The higher H_2_ yield associated with butyrate production (reaction c) will inhibit NADH oxidation more than propionate production (reaction d), which explains why propionate production is more upregulated than butyrate production at increased *P*_H_2__. Furthermore, production of butyrate yields only one VFA per equivalent of glucose (reaction c), whereas production of acetate and propionate (reaction d) yields two VFA per equivalent of glucose, which makes the rumen environment more acidic. Shortly after a meal, propionate may be produced via lactate production, via reaction e. Lactate is a stronger acid than propionate and makes the rumen environment even more acidic. In addition to the net 0.67 NADH oxidized back to NAD^+^, the acidic environment promotes the oxidation of NADH. Less inhibition of NADH oxidation at lower pH (Fig 2) explains why, at neutral or alkaline pH, propionate production is more effective in maintaining the NAD^+^ to NADH ratio than butyrate production [9].

Thermodynamic control of *P*_H_2__ on NADH oxidation but not on the glucose fermentation pathways, is also in line with the statement that the NAD^+^ to NADH ratio determines the profile of VFA produced with rumen fermentation [2]. One may designate this as the dynamic control of *P*_H_2__ on rumen fermentation pathways. Ghimire et al. [12], building on the Molly cow model, which includes a representation of rumen fermentation processes, attempted to account for the effect of the thermodynamic state of the rumen environment on the interconversion between acetate and propionate. Besides keeping *P*_H_2__ constant in the calculation of these rate constants, they did not consider the NAD^+^ to NADH ratio, which might have caused their model not to perform well in predicting observed variation in ruminal VFA production. Future modeling attempts might benefit from a representation of the NAD^+^ to NADH ratio.

Even though an empirical relationship between *P*_H_2__ and proportion at which individual VFA are produced may appear from experimental data, the validity of a NAD-driven mechanistic prediction of metabolic end products is supported by the work of Salem et al. [47]. They used the NAD^+^ to NADH ratio as a key controller of the type of glucose degradation products to be formed. Although their modeling effort deals with the myocardial energy metabolism, which partly differs from the energy metabolism of anaerobic bacteria, a similar approach may be applied for estimating rumen fermentation products. Oxygen concentration in blood, like *P*_H_2__ in anaerobic environments, dictates redox conditions and consequently the NAD^+^ to NADH ratio. Therefore, predicting the production of individual VFA in the rumen might benefit from using the NAD^+^ to NADH ratio as a controlling factor as was suggested from an evaluation of various VFA prediction models [48]. Future modeling attempts might benefit from a representation of the NAD^+^ to NADH ratio.

The NAD^+^ to NADH ratio as a key controller of the type of VFA produced explains why feeding rapidly degradable carbohydrates induces a shift from acetate to propionate production in the rumen. This shift has been confirmed by various studies, among which a regression analysis of molar proportions of VFA production [6] and a metabolic model of mixed culture fermentations [9, 46]. Different carbohydrate polymers such as cellulose and amylose are broken down to the same monomers, and can be converted into the same fermentation end products. Degradation rate of carbohydrates, however, determines the magnitude of the increase in *P*_H_2__ and decrease in NAD^+^ to NADH ratio obtained via the glycolysis, which controls pathways of VFA production from pyruvate. The ability of specific microbial species to catalyze the breakdown of a certain type of carbohydrate polymer might be related to the production of specific VFA, like starch hydrolysis favors propionate production. Nonetheless, this may also be regarded as the NAD^+^ to NADH ratio controls fermentation pathways, where the metabolic physiology of these species has been adapted to degrade specific carbohydrate polymers in the rumen.

### VFA interconversion

Interconversion of VFA in the rumen has been discussed various times in the literature [10, 12]. After measuring VFA production rates in the rumen of lactating dairy cows by infusion of ^14^C labeled VFA, all six possible conversions between acetate, propionate and butyrate were confirmed to occur [49]. Of these conversions, acetate to propionate, propionate to acetate and butyrate to acetate are H_2_-dependent.

**Fig 3:**
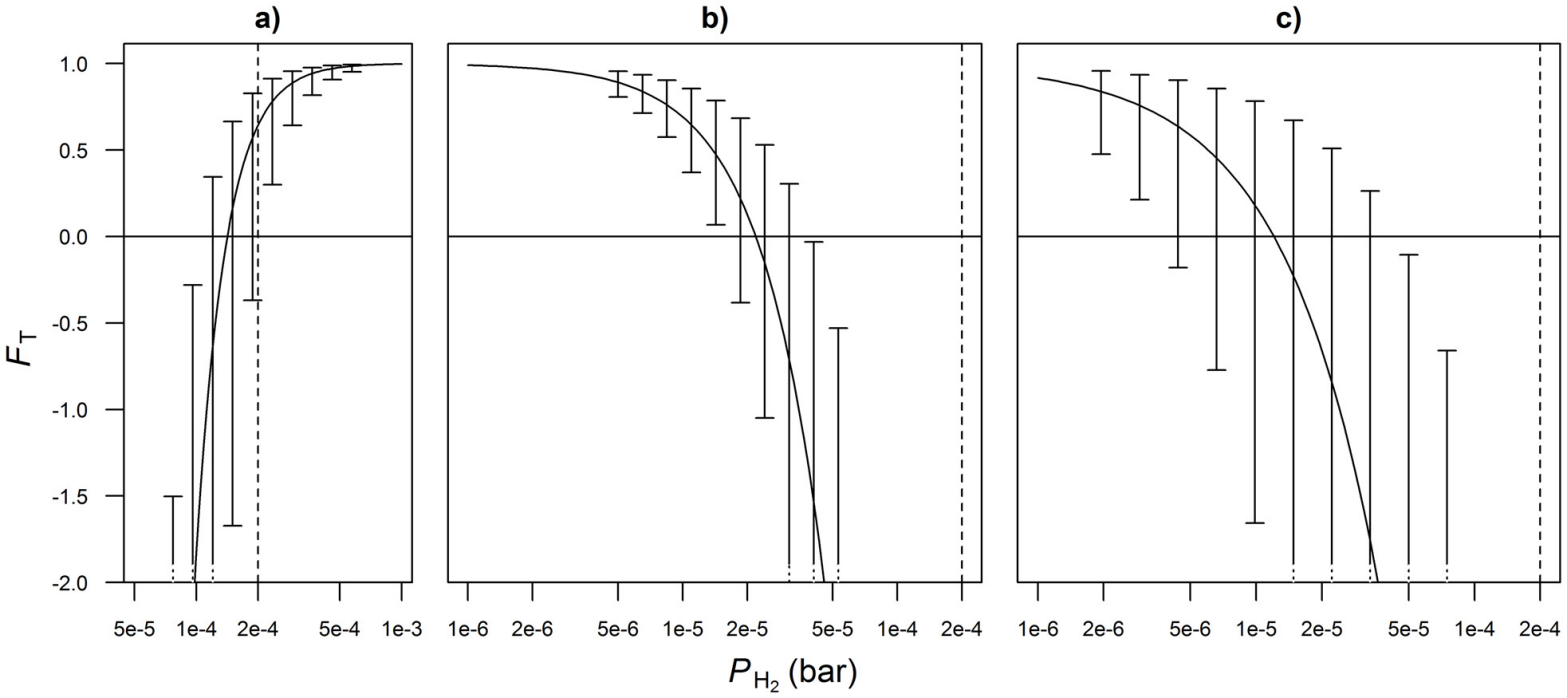
Thermodynamic potential factor (*F*_T_) as a function of *P*_H_2__ for VFA interconversions. Conversions comprise a) acetate to propionate, b) propionate to acetate, c) butyrate to acetate. The 95% confidence intervals represent uncertainty of *F*_T_ to variation in inputs other than *P*_H_2__. Vertical lines demarcate the rumen physiological lower bound of *P*_H_2__. A log scale is used to plot the x-axis.

Acetate to propionate conversion was observed at 2.0% and 2.6% of de novo synthesized acetate being converted into propionate at normal and low-roughage diets, respectively [49]. The higher conversion rate from the low-roughage diets may be attributed to higher *P*_H_2__ from the more rapidly degradable carbohydrates. To the authors’ knowledge, there is only one study that has described this conversion [15]. Therein, H_2_-dependent propionate production from acetate and CO_2_ by a pure culture of *Desulfobulbus propionicus* was reported. This particular study focuses on freshwater sediments and other microbial species might be responsible for this conversion in the rumen. The *F*_T_ for this reaction increased from zero to unity for *P*_H_2__ between approximately 1.5⋅10^−4^ and 5⋅10^−4^ bar, and zero is no longer within the confidence interval of *F*_T_ for *P*_*H*_2__ > 2.3⋅10^−4^ bar (Fig 3a), implying the conversion of acetate to propionate to be controlled by *P*_H_2__ and thermodynamically feasible under common rumen conditions. However, Laanbroek at al. [15] also reported not having observed any propionate from acetate and CO_2_ in the presence of sulfate. Traces of sulfate may enter the rumen with regular feedstuffs and will be metabolized by the microbes [50]. Especially when diets contain co-products from grain milling industries rumen sulfate concentrations may be high. Apart from *P*_H_2__, also the sulfate concentration might control the rate of conversion of acetate to propionate. Besides, sulfate is an electron acceptor for respiration and will also compete for electrons and lower CH_4_ production [51].

The H_2_-dependent conversions of propionate and butyrate into acetate yield multiple equivalents of H_2_ (reactions g and h, Table 1) and require very low *P*_H_2__ to make them exergonic and proceed. For both reactions, values of *F*_*T*_ ≥ 0 are within the 95% confidence interval for *P*_*H*_2__ < 4⋅10^−5^ bar (Fig 3b and 3c). This indicates these conversions do not occur under conditions that are common in the rumen where *P*_H_2__ is usually higher. However, propionate or butyrate degrading bacteria may aggregate with H_2_-consuming methanogens in typical syntrophic associations. This association of cells enables interspecies H_2_ transfer by diffusion, and its flux is enhanced when the intermicrobial distance decreases [4, 52]. If this local interspecies H_2_ transfer occurs, *P*_H_2__ is lower than in other locations of the rumen, which makes the oxidation of propionate and butyrate exergonic. Furthermore, sulfate- and nitrate-reducing conditions have been reported to thermodynamically favor the degradation of propionate and butyrate [52]. Degradation of VFA under these conditions is H_2_-independent, as was reported for propionate to acetate conversion in the presence of sulfate [15]. Therefore, the inhibition of *P*_H_2__ on the butyrate to acetate and propionate to acetate conversions might be counteracted in the presence of external electron acceptors. These conversions, though, require microbes capable of respiration.

Another pathway involving propionate to acetate conversion was described by De Bok et al. [53]. Using ^13^C labeled compounds, they found *Smithella propionica* to convert propionate into acetate and butyrate via a six-carbon intermediate. This particular conversion of propionate also gives physiological evidence for the conversion of propionate into butyrate. Hydrogen is not directly involved in this pathway and indicates the conversion of propionate into either acetate or butyrate is not affected by *P*_H_2__. Depending on the *P*_H_2__, the concentrations of acetate, propionate and butyrate, and the abundance of microbial aggregates, this particular propionate conversion into acetate and butyrate may enable butyrate oxidation in methanogenic ecosystems in case the classical propionate oxidation pathway would be endergonic [54]. In other words, this makes sense for the range of *P*_H_2__ with *F*_*T*_ > 0 for butyrate oxidation and *F*_*T*_ < 0 for propionate oxidation. This range is negligibly small and below 2⋅10^−4^ bar (Fig 3b and 3c), explaining why this particular oxidation of propionate is not expected to occur under rumen conditions.

Besides the VFA interconversions discussed in the paragraphs above, the acetate to butyrate conversion is ecologically significant [55] and seems to be more substantial than the other VFA interconversions in the rumen [49]. The final metabolic step of butyrate production, butyryl-CoA to butyrate, proceeds via butyrate kinase or via butyryl-CoA:acetate CoA-transferase [55]. Acetate to butyrate conversion may be described by the latter mechanism. For this conversion, apart from acetate, another substrate such as hexose is required to yield butyryl-CoA. From human colon microbiota, genes encoding for enzymes for both pathways were detected in various *Butyrivibrio fibrisolvens* strains and *Clostridium* species that also reside in the rumen. The butyryl-CoA:acetate CoA-transferase step does not yield H_2_ and will not be affected by *P*_H_2__. The *F*_T_ for the conversion of glucose and acetate to butyrate did not deviate from unity for *P*_H_2__ between 2⋅10^−5^ and 5⋅10^−2^ bar. Furthermore, for butyrate formed via both butyryl-CoA:acetate CoA-transferase and butyrate kinase, butyryl-CoA is formed from pyruvate with the same metabolic steps. Hence, the two mechanisms of butyrate production yield the same H_2_ balance and oxidize equal equivalents of NADH to NAD^+^ per equivalent of glucose (reaction c, Table 1; [3, 56]. Butyrate production via butyryl-CoA:acetate CoA-transferase and via butyryl kinase are therefore not controlled differently by the NAD^+^ to NADH ratio and *P*_H_2__. This would make a specific *P*_H_2__-controlled flux of acetate to butyrate conversions in rumen dynamic modeling efforts redundant.

The ecological significance of the conversion of butyrate to propionate is low. Because Δ*G*^*o*^ for the propionate conversion into acetate and butyrate is nearly zero [57], the reverse reaction from butyrate to propionate might occur too. Furthermore, the metabolism of threonine fermentation in *Clostridium propionicum* has been described to yield both propionate and butyrate via 2-oxobutyrate [58]. The conversion of butyrate into propionate might occur as a side reaction, albeit the actual occurrence via 2-oxobutyrate is questionable.

The different fluxes of rumen VFA in the three-pool model of Sutton et al. [49] suggests that accounting for *P*_H_2__ controlled VFA interconversions in dynamic model predictions is compatible with the conversions of acetate to propionate, butyrate to acetate and propionate to acetate. Nonetheless, these VFA interconversions are still controlled by the NAD^+^ to NADH ratio of which the dynamics, described in the present investigation, may already explain an important part of the observed variation in the proportion of individual VFA. Prediction of VFA interconversion would also require information such as intermicrobial distance in syntrophic aggregates and concentration of external electron acceptors such as nitrate and sulfate. Including this information in a model next to control by NAD^+^ to NADH ratio increases the model complexity, and it needs to be further investigated whether it aids in explaining observed variation in the proportion of individual VFA. Furthermore, functions that microorganisms carry out in certain experimental settings may differ greatly, depending on the presence or absence of other community members [42]. Applying this differing of functions to VFA interconversions makes dynamic predictions of rumen VFA concentrations uncertain.

### Methanogenesis

The *F*_T_ for methanogenesis increased from zero to unity for *P*_H_2__ at ∼ 10^−5^ bar for archaea without cytochromes and at ∼ 10^−3^ bar for archaea with cytochromes (Fig 1). This indicates a certain threshold of *P*_H_2__ to make methanogenesis proceed, depending on the physiology of the archaea. For methanogenesis by archaea with cytochromes, *F*_*T*_ = 0 for *P*_*H*_2__ ≈ 3⋅10^−3^ bar and and based on the 95% confidence interval *F*_*T*_ ≤ 0 for *P*_*H*_2__ < 8⋅10^−4^ bar (Fig 1). Rumen *P*_H_2__ may be as low as 2⋅10^−4^ bar [2] which will yield a negative *F*_T_ and may explain why archaea with cytochromes are hardly found in the methanogenic community in the rumen [23, 59]. Given that *F*_T_ approaches unity with rather minor uncertainty at *P*_H_2__ as low as 2⋅10^−4^ bar (Fig 1), methanogenesis by archaea without cytochromes is hardly restricted by the thermodynamic state of the rumen environment.

The amount of H_2_ present in the rumen has been expressed as dissolved H_2_ concentration [11]. It is common to express gas contents in pressure, but the possible occurrence of supersaturation of dissolved H_2_ (e.g., [60]) would necessitate the use of dissolved H_2_ concentration instead of *P*_H_2__. Supersaturation, the violation of Henry’s Law, is the non-equilibrium condition between dissolved H_2_ concentration and *P*_H_2__ in the rumen headspace. The fact that archaea with cytochromes hardly exist in the rumen might suggest too low dissolved H_2_ concentrations for their survival and negligible supersaturation of H_2_. Furthermore, rumen contractions may prevent supersaturation of H_2_ to occur. If supersaturation does occur in the rumen, survival of archaea with cytochromes may be enabled and the NAD^+^ to NADH ratio may become lower than indicated in the present study.

Several studies have recognized the importance of adequate coefficients of production rate of individual VFA to accurately predict CH_4_ [7, 8, 12]. The present finding that, under common rumen conditions, VFA dynamics rather than methanogenesis is controlled by *P*_H_2__, confirms that the thermodynamic control on the type of VFA formed is significant and should be further elaborated. This finding corresponds with conclusions in previous publications [10, 11]. In contrast to these studies, however, it is argued here that the NAD^+^ to NADH ratio should be considered as a key controller of the type of VFA produced and the associated amount of H_2_ being formed available for methanogenesis, as also described in ref. [2]. The present theoretical effort, indicates that taking the NAD^+^ to NADH ratio into account in dynamic rumen models is likely to improve prediction of type of VFA formed and CH_4_ emissions.

It is concluded that fermentation of glucose to various VFA proceeds far from thermodynamic equilibrium and is not controlled by *P*_H_2__ under rumen physiological conditions. However, oxidation of NADH does appear to be controlled by *P*_H_2__, where the actual control also depends on the intracellular pH of microorganisms and the involvement of ferredoxin in NADH oxidation. The conversion of acetate to propionate is thermodynamic controlled by *P*_H_2__ and also depends on the NAD^+^ to NADH ratio. Conversions of butyrate to acetate and propionate to acetate are thermodynamically suppressed by *P*_H_2__ and will not proceed without aggregation of rumen microbes. Rumen methanogenesis by archaea without cytochromes, which comprise most of the methanogenic population in the rumen, appears not to be thermodynamically restricted by *P*_H_2__, implying the thermodynamic control of *P*_H_2__ to be negligible. Representation of the key role of the NAD^+^ to NADH ratio in rumen fermentation models is required to improve the accuracy of prediction of VFA and CH_4_ production by these models.

## Supporting Information

S1 File R code for calculating 95% confidence intervals of the thermodynamic potential factor (*F*_T_) at discrete values of *P*_H_2__ and for plotting of *F*_T_ as a function of *P*_H_2__, including the 95% confidence intervals, for glucose fermentation and methanogenesis (Fig 1). (R)

S2 File R code for plotting of the thermodynamic potential factor (*F*_T_) as a function of *P*_H_2__ for NADH oxidation (Fig 2). (R)

S3 File R code for calculating 95% confidence intervals of the thermodynamic potential factor (*F*_T_) at discrete values of *P*_H_2__ and for plotting of *F*_T_ as a function of *P*_H_2__, including the 95% confidence intervals, for VFA interconversions (Fig 3). (R)
