# Correction: Thermodynamic Driving Force of Hydrogen on Rumen Microbial Metabolism: A Theoretical Investigation

**DOI:** 10.1371/journal.pone.0168052

**Published:** 2016-12-09

**Authors:** Henk J. van Lingen, Caroline M. Plugge, James G. Fadel, Ermias Kebreab, André Bannink, Jan Dijkstra

The affiliation for the fourth author is incorrect. Ermias Kebreab is not affiliated with #3 but with #4 The Department of Animal Sciences, University of California, Davis, Davis, California, United States of America.
